# Supplementary material for: DMRforPairs: identifying Differentially Methylated Regions between unique samples using array based methylation profiles
Source: BMC Bioinformatics. 2014 May 15;15:141. doi: 10.1186/1471-2105-15-141 (PMC4046028; doi:10.1186/1471-2105-15-141)
Supplement: Additional file 2 — DMRforPairs output for the comparison of A431-MCF7 and NA17018-NA17105. Please start from the HTML files in each folder. Available via the BMC Bioinformatics website. [file 1471-2105-15-141-S2.zip › 1394847754114233_MOESM2_ESM/A431_MCF7/figures/16740.pdf]

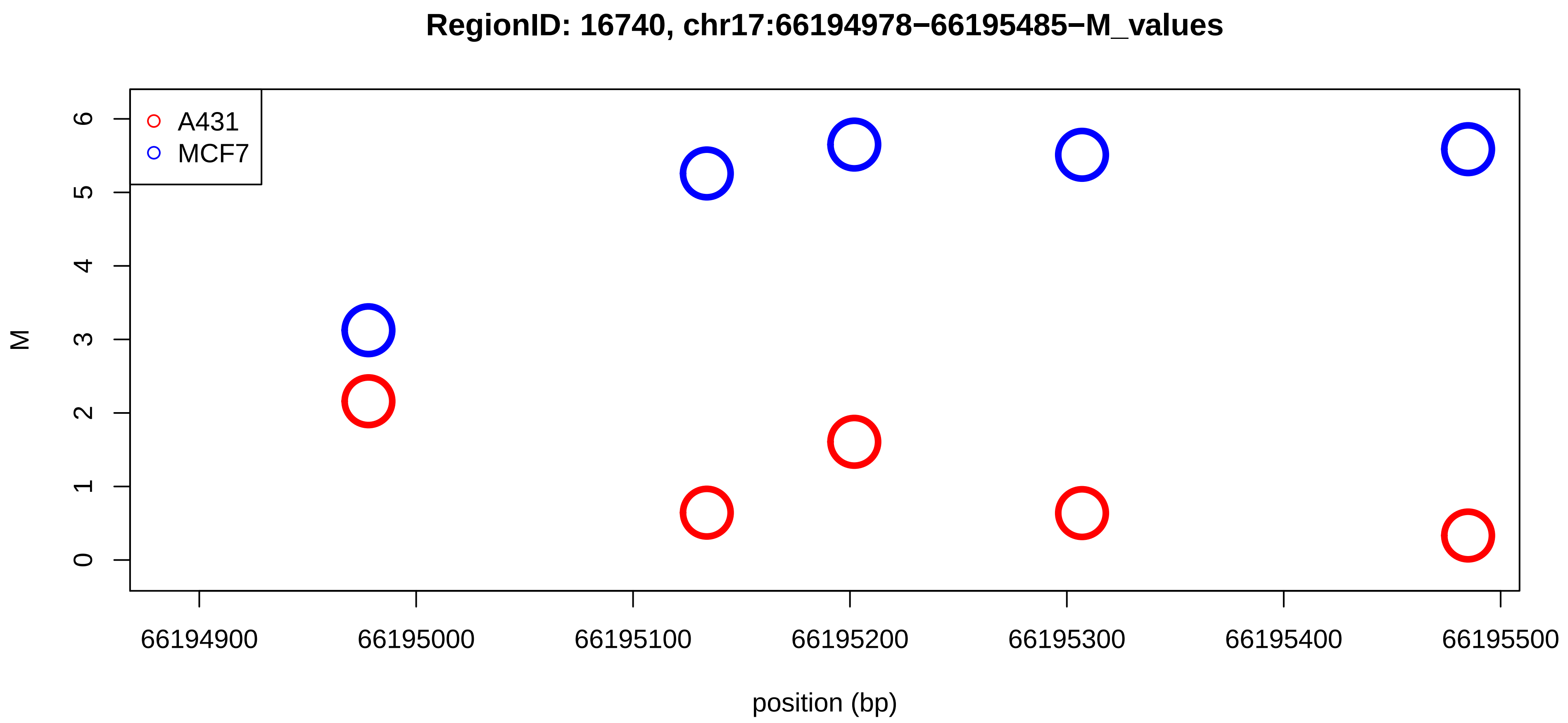
RegionID: 16740, chr17:66194978−66195485−M_values
A431
MCF7
M
0
1
2
3
4
5
6
66194900
66195000
66195100
66195200
66195300
66195400
66195500
position (bp)

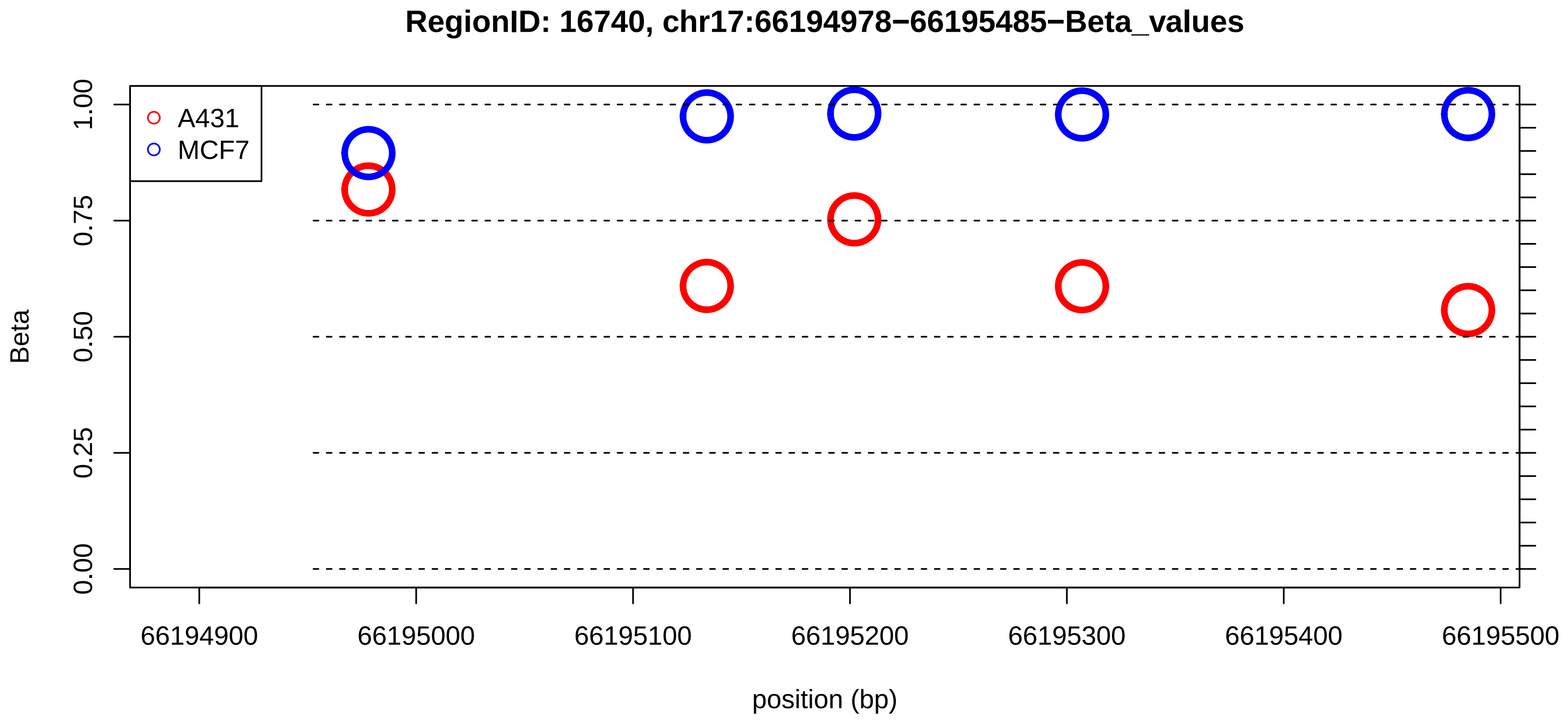
RegionID: 16740, chr17:66194978−66195485−Beta_values
A431
MCF7
Beta
0.00
0.25
0.50
0.75
1.00
66194900
66195000
66195100
66195200
66195300
66195400
66195500
position (bp)

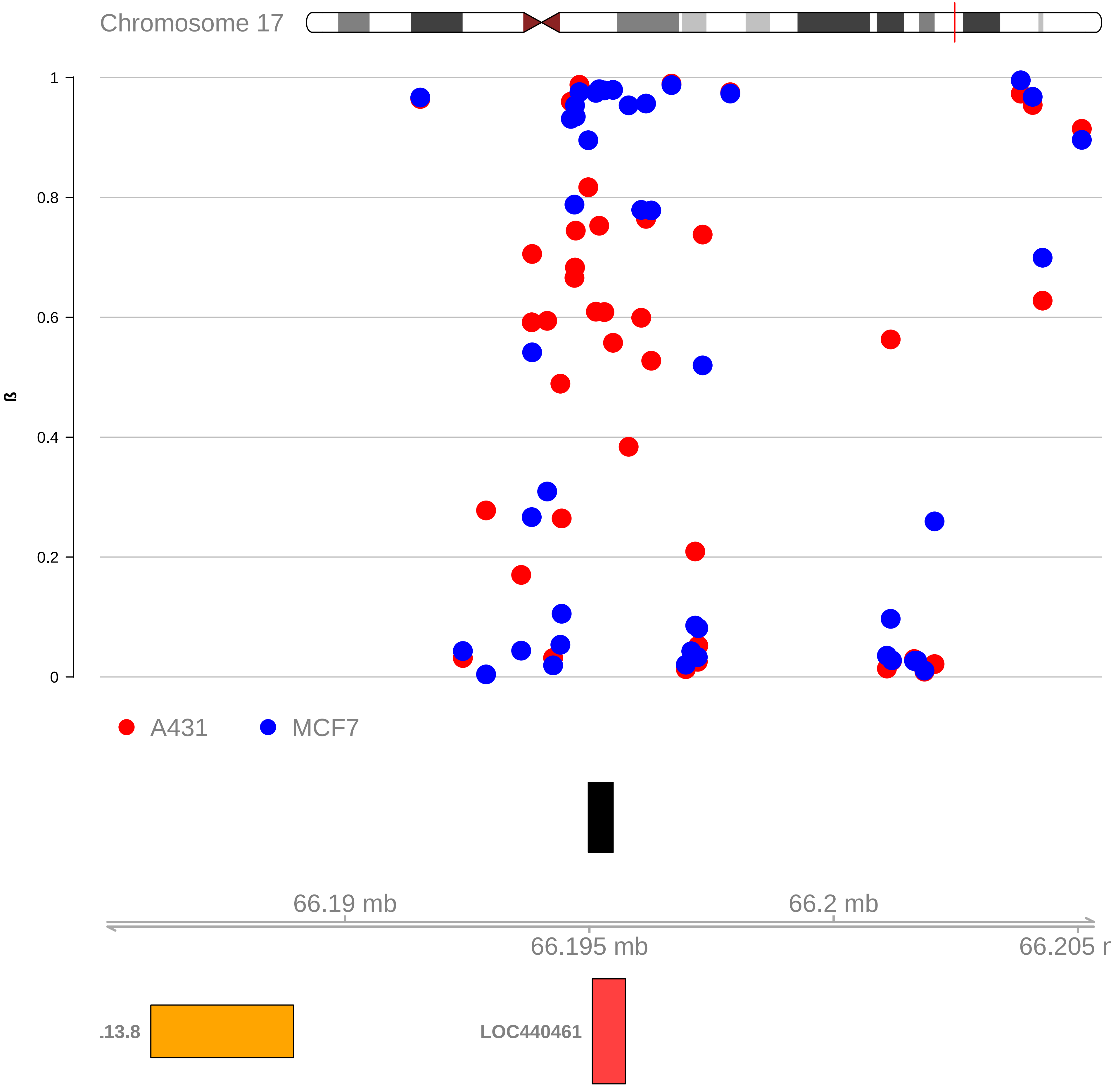
Chromosome 17
1
0.8
0.6
0.4
0.2
0
ß
A431
MCF7
66.19 mb
66.2 mb
66.195 mb
66.205 m
.13.8
LOC440461
